# Shoshin Beriberi and Severe Accidental Hypothermia as Causes of Heart Failure in a 6-Year-Old Child: A Case Report and Brief Review of Literature

**DOI:** 10.3389/fped.2019.00119

**Published:** 2019-03-29

**Authors:** Alfredo Vicinanza, Corinne De Laet, Shancy Rooze, Ariane Willems, Xavier Beretta-Piccoli, Daphné Vens, Cédric Voglet, Caroline Jacquemart, Martial Massin, Dominique Biarent

**Affiliations:** ^1^Pediatric Intensive Care Department, Hôpital Universitaire des Enfants Reine Fabiola, Université Libre de Bruxelles, Brussels, Belgium; ^2^Nutrition and Metabolic Unit, Hôpital Universitaire des Enfants Reine Fabiola, Université Libre de Bruxelles, Brussels, Belgium; ^3^Division of Pediatric Cardiology, Hôpital Universitaire des Enfants Reine Fabiola, Université Libre de Bruxelles, Brussels, Belgium

**Keywords:** severe accidental hypothermia, heart failure, malnutrition, thiamine deficiency, refeeding syndrome, child

## Abstract

Severe accidental hypothermia has been demonstrated to affect ventricular systolic and diastolic functions, and rewarming might be responsible of cardiovascular collapse. Until now, there have been only a few reports on severe accidental hypothermia, none of which involved children. Herein, we describe here a rare case of heart failure in a 6-year-old boy admitted to the emergency unit owing to severe hypothermia and malnutrition. After he was warmed up (core temperature of 27.2°C at admission), he developed cardiac arrest, requiring vasoactive amines administration, and veno-arterial extracorporeal membrane oxygenation. Malnutrition and refeeding syndrome might have caused the thiamine deficiency, commonly known as beriberi, which contributed to heart failure as well. He showed remarkable improvement in heart failure symptoms after thiamine supplementation. High-dose supplementation per os (500 mg/day) after reconstitution of an adequate electrolyte balance enabled the patient to recover completely within 2 weeks, even if a mild diastolic cardiac dysfunction persisted longer. In conclusion, we describe an original pediatric case of heart failure due to overlap of severe accidental hypothermia with rewarming, malnutrition, and refeeding syndrome with thiamine deficiency, which are rare independent causes of cardiac dysfunction. The possibility of beriberi as a cause of heart failure and adequate thiamine supplementation should be considered in all high-risk patients, especially those with malnutrition. Refeeding syndrome requires careful management, including gradual electrolyte imbalance correction and administration of a thiamine loading dose to prevent or correct refeeding-induced thiamine deficiency.

## Introduction

The effects of severe hypothermia and rewarming on human cardiac function have not yet been well established, especially in children. However, it is known that hypothermia may play a role in cardiac dysfunction (1, 2). Another cause of heart failure, especially in intensive care, is thiamine deficiency (TD). Despite the fact that it could be easily treated, TD continues to occur all age groups in both high and low resource countries with potentially severe and life-threatening consequences, including cardiovascular “shoshin” beriberi (3). Since children with malnutrition may have low or borderline thiamine reserves, they are prone to developing TD during refeeding without thiamine supplementation (4).

## Case Description

We report a case of a 6-year-old boy admitted to the emergency unit owing to severe hypothermia and unconsciousness. Medical history was not contributive.

He was transferred by a mobile emergency medical service to the hospital emergency room. Vital parameters and clinical examination at admission showed a Glasgow Coma Scale score of 5/15 with fixed dilated pupils, a core rectal temperature of 27.2°C, SpO_2_ of 98% with supplemental oxygen, irregular respiration, sinus bradycardia (60 bpm), a normal blood pressure (98/72 mmHg, mean blood pressure 79 mmHg), a prolonged capillary refilling time of 4 s, muscle rigidity, and obvious hypotrophy.

During admission, rapid-sequence intubation and non-invasive rewarming were performed. Fluid replacement boluses of normal saline were administered. Blood gas and first laboratory assessments within 8 h of admission showed a compensated metabolic acidosis, hyperlactatemia [Lactatemia at 5.5 mmol/L, normal (*N*): <2 mmol/L], hyperglycemia at 340 mg/dL (*N*: 70–100 mg/dL), mild thrombocytopenia (124,000/μL, *N*: 150,00–440,000/μL), elevated biomarkers of myocardial and muscular damage such as serum CK-MB (up to 2,262 UI/L, *N*: <190 UI/L), myoglobin (up to 1,978 ng/mL, *N*: <72 ng/mL), and troponin (up to 684 ng/L, *N*: <14.0 ng/L), mild alteration of coagulation screening [(PT 51% (*N*: >70%), INR 1.46 (*N*: 0.95–1.31)], hypertransaminasemia (AST up to 2,700 UI/L, *N*: < 40 UI/L, ALT up to 2,000 UI/L, *N*: <41 UI/L), normal renal and pancreatic functions, and no major ionic problems. Electrocardiography showed a first-degree atrioventricular block, with a prolonged QTc interval at 470 ms.

**Figure 1:**
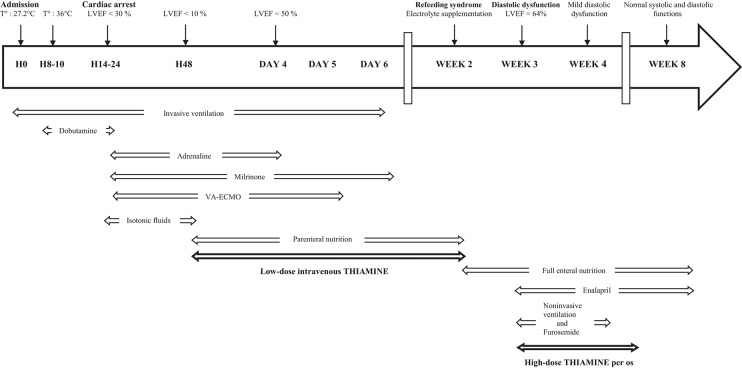
Timeline of the events. H, hour; T°, core rectal temperature; LVEF, left ventricular ejection fraction; VA-ECMO, veno-arterial extracorporeal membrane oxygenation.

He was admitted to the pediatric intensive care unit (at H0 = hour 0) and his core temperature was restored (up to 36°C) over 8 h. Persistent hyperlactatemia justified initiation of dobutamine infusion (5 μg/kg/min). Six hours after the patient was warmed up (H14), a cardiogenic shock with cardiac arrest (non-perfusing rhythm—pulseless electrical activity) suddenly occurred and a return of spontaneous circulation was obtained within 30 min of cardio-pulmonary resuscitation performed according to the European guidelines (5). No arrhythmias occurred. Echocardiography showed a structurally normal heart with a biventricular global dysfunction [left ventricular ejection fraction (LVEF) of 30%, with mitral insufficiency of grade 2/4 and protodiastolic and end-diastolic pressures, measured on pulmonary insufficiency, at 22 and 15 mmHg, respectively]. At that moment, the serum NT-pro-BNP level was 1,195 pmol/L (*N*: <15 pmol/L). Hemodynamic support with adrenaline [maximum (Max) dose of 0.3 μg/kg/min] and milrinone (Max dose 0.375 μg/kg/min) infusions was initiated. Dobutamine was stopped. Despite the use of amines, degradation of mainly the left heart function occurred within a few hours (H20), requiring veno-arterial extracorporeal membrane oxygenation (VA-ECMO). At H48, the cardiac function was still worsening (LVEF <10%) (Figure 1). Other organ injuries, sepsis, intoxication, adrenal insufficiency, and thyroid dysfunction were excluded.

A nutritional screening performed at admission before VA-ECMO support in this context of maltreatment showed a pre-albumin at 0.07 g/L (*N*: 0.24–0.40 g/L), an albumin of 31 g/L (*N*: 38–54 g/L), and multivitamin deficiencies (Vitamin A, 0.34 μmol/L, *N*: 0.87–2.62 μmol/L; Vitamin D, 15.2 nmol/L, *N*: 75.0–150.0 nmol/L; Vitamin E, <1.16 μmol/L, *N*: 11.60–41.79 μmol/L), indicating a state of malnutrition. Serum zinc and copper levels were at the lowest limits of the normal range. Serum selenium level was low at 44 μg/L (*N*: 50–150 μg/L). Serum free and total carnitine levels were within the normal range. An abnormal iron balance was highlighted too. Moreover, measurement of anthropometric parameters at admission, showing a height for age *z*-score at −2.1, confirmed the clinical state of chronic malnutrition.

Total parenteral nutrition with vitamin supplementation (thiamine nitrate 2.5 mg/day) was therefore started at H48. Before starting parenteral nutrition, the patient was fasted with only isotonic intravenous fluids and no enteral feeding. The biventricular function dramatically improved and allowed us to wean off invasive ventilation, amines and extracorporeal assistance within day 6 (Figure 1). Echocardiography performed at day 4 showed an LVEF of 50%. Total enteral nutrition was achieved 1 week after stopping VA-ECMO (Figure 1).

A few days after starting full enteral nutrition, despite avoiding rapid increases in the daily caloric intake, a refeeding syndrome appeared (hypokalemia at 2.7 mmol/L, *N*: 3.5–4.8 mmol/L; hypomagnesemia at 0.49 mmol/L, *N*: 0.66–1.07 mmol/L; hypophosphatemia at 0.28 mmol/L, *N*: 1.02–1.86 mmol/L), which required electrolyte supplementation, almost concomitant with acute pulmonary edema, stiff hepatomegaly, and other signs of pre-charge overload. Thoracic computed tomography showed dilatation of the left atrium with high serum NT-pro-BNP (1,395 pmol/L). Echocardiography at week 3 showed a diastolic dysfunction (proto-diastolic pressure estimated at 25 mmHg, moderate dilatation of the inferior vena cava) despite a normal systolic function with LVEF of 64%. Non-invasive ventilation, furosemide, and enalapril were started. Given that “wet beriberi” was suspected despite the absence of biological confirmation (vitamin B1 dosage was not available), supplementation of high-dose thiamine was initiated (500 mg/day per os). Clinical improvement was rapidly obtained within a few days. Despite a complete recovery of the systolic heart function, a mild diastolic dysfunction persisted. One month later both systolic and diastolic functions normalized (Figure 1).

Moreover, the patient underwent a depressive-like state suggestive of post-traumatic stress. Wernicke-like syndrome was excluded. No nystagmus, ophtalmoplegia, or ataxia were noted, and brain MRI findings were normal. His behavior progressively and completely returned to a normal state. The patient's condition continued to improve and 1 month later, the patient's clinical and biochemical parameters were also completely restored.

## Discussion

To our knowledge, we described the first case of suspected beri-beri and hypothermia-induced heart failure in a child.

It has been shown that hypothermia below 34°C significantly reduces cardiac contractile functional variables (1). Although the occurrence of life-threatening cardiac arrhythmias usually subside with increasing core temperature, hypotension, and low cardiac output may prevail during and after rewarming (1). It has been demonstrated that post-hypothermic contractile dysfunction is due to an isolated perturbation of systolic function, whereas diastolic function is restored (1). More recently, accidental hypothermia has been reported to affect left ventricular systolic and diastolic functions in adults (2). However, there are only a few reports concerning severe accidental hypothermia (core temperature below 28°C), but none in children, and most of the data are derived from animal experimental studies (2). Although the complete pathophysiology of hypothermia-induced cardiac failure is not known, it seems that, among other factors, cytosolic Ca^2^^+^ overload is involved, possibly via a temperature-dependent dysfunction of ion transport (6). The excitation-contraction coupling and the actin-myosin interaction may be persistently depressed despite normal body temperature (7).

It is also known that in moderate and severe hypothermia circulating blood volume is reduced (8). During rewarming, vasoconstriction that previously limited the vascular space was eliminated, which led to cardiovascular collapse within 6 h after the patient was warmed to achieve a normal core temperature. Our patient did not present any life-threatening arrhythmias during severe hypothermia, but his left ventricular systolic function severely deteriorated a few hours after he reached 36°C. Moreover, his diastolic function was also altered.

A recent study showed that parameters of left ventricular systolic function were significantly reduced in patients with protein energy malnutrition as compared to that in controls (9). Starvation and food deprivation caused our patient's malnutrition, as confirmed by his anthropometric and biochemical evaluation findings, which could have increased his systolic dysfunction.

Malnutrition is associated with an increased risk of B-complex vitamin deficiency, namely thiamine deficiency (10). Regarding the essential micronutrients, the body's requirements are exclusively dependent on the dietary supply, as these micronutrients could not be endogenously synthesized. The combination of limited body storage and a high turnover rate (half-life <10 days) could result in potential depletion of thiamine stores within 2 weeks if it is not continuously replaced (3). This can be particularly troublesome for malnourished and critically ill patients (4), similar to our case. Thiamine deficiency has been shown to be present in patients upon intensive care unit (ICU) admission and can develop over time in ICU patients (10). They are also prevalent in patients with septic shock and other critically ill conditions, with rates ranging from 10 to 70% (11). Thiamine (vitamin B1) is a cofactor of key metabolic enzymes (such as pyruvate dehydrogenase), and TD may cause alteration in heart metabolism and has been reported to cause heart failure (12).

Deficiency in thiamine prevents the ability of blood vessels to vasoconstrict, which leads to decreased systemic vascular resistance (SVR) and systemic blood pressure. Although cardiovascular beriberi is classically thought to represent a high-output state to compensate for decreased SVR (13), beriberi less commonly presents as fulminating heart failure with evidence of low cardiac output, vascular collapse, and lactic acidosis (shoshin beriberi) (3, 14). Without treatment this condition can progress into severe acute congestive heart failure, edema, respiratory distress, and eventually death within a few days (3). The precise mechanism remains unknown, but direct injury of the myocardium by TD may play a critical role (13).

The presence of a depression of the left ventricular function has been observed during the acute phase of beriberi heart disease. In addition to beriberi heart disease, coexistent diseases may contribute to relative depression of the left ventricular function, leading to acute decompensation, probably following the increased rate of cellular metabolism after rewarming. We hypothesized that the initial cardiac dysfunction in our case could have been sequelae of a rewarmed heart with a non-existent cardiac reserve secondary to thiamine deficiency. The selenium deficiency might have played a role in the clinical severity of heart failure as well.

Previous studies have shown the disposition of drugs to be affected during ECMO, probably as a result of the expanded circulating volume and interactions with the polymeric components of the extracorporeal circuit (15). The ECMO circuit may sequester a variety of circulating compounds, such as drugs and possibly nutrients, effectively reducing the bioavailability of these compounds (16). The start of ECMO with its consequent increased circulatory volume could have resulted in critical reduction of thiamine levels as well.

Our patient showed a remarkable improvement in congestive heart failure symptoms.

Fresh frozen plasma was added to the ECMO circuit to prevent or correct abnormal coagulation. We cannot exclude that plasma could have counterbalanced the fall in oncotic pressure due to loss of intravascular proteins during rewarming.

The ECMO itself and potentially the added plasma could have contributed to the patient's improvement as well as the initiation of low-dose supplementation of intravenous thiamine, even if a low serum thiamine level has not been confirmed. However, in the absence of specific diagnostic tests and real-time laboratory data, the only way to diagnose TD is to carry out a therapeutic thiamine challenge (3).

Although all vitamin deficiencies may occur at variable rates with inadequate intake, TD is the most important complication of refeeding (17). Refeeding syndrome can be defined as the potentially fatal shifts in fluids and electrolytes that may occur in malnourished patients receiving artificial refeeding (whether enterally or parenterally) (17). During refeeding, glucose-involved insulin secretion causes abrupt switch from catabolism to anabolism. This creates a sudden cellular demand for electrolytes (phosphate, potassium, and magnesium) necessary for synthesis of adenosine triphosphate, glucose transport, and other synthesis reactions, resulting in decreased serum levels and severe electrolyte imbalances (18). In induced TD, refeeding stimulates insulin production leading to protein synthesis and increased thiamine demand due to glucose utilization. Pre-existing low thiamine levels are further reduced by this increased cellular consumption of thiamine (3). Moreover, there is experimental evidence that magnesium may be necessary for complete thiamine utilization (14). Furthermore, volume overload begins with an increase in insulin secretion during the early stage of refeeding the patient. This eventually increases renal sodium reabsorption and retention, and then fluid retention (19).

The mechanism of the cardiovascular impairment that occurred in our patient during refeeding is probably multifactorial. We speculated that our patient was being supplemented with insufficient amounts of intravenous thiamine to restore his deficiency while on parenteral nutrition. Despite a very careful introduction of feeds, refeeding syndrome has been triggered. Consequently, the co-existing increased thiamine demand and its reduced utilization due to metabolic changes and hypomagnesemia respectively, along with fluid overload, might have contributed to the patient's congestive heart failure. High-dose thiamine supplementation per os (500 mg/day) after reconstitution of an adequate electrolytes' balance enabled patient's complete recovery within 2 weeks.

Furthermore, it has been postulated that bi-ventricular diastolic dysfunction after severe accidental hypothermia persists on discharge from the ICU despite the recovery of systolic function in survivors after ECMO rewarming (2). On the basis of these findings, the persistent alteration of the cardiac diastolic function in our patient could also be due to sequelae of hypothermia.

Given that beriberi clinical features are non-specific for TD diagnosis, a policy recommendation for empirical therapy with thiamine would be justified for sick children living in endemic areas and those patients who are critically ill and at risk of TD. No tolerable upper intake level has been set for thiamine. In severe deficiency states, oral and intravenous administrations of doses far above the daily recommended intake for healthy people are considered safe (4).

## Concluding Remarks

To the best of our knowledge, this was the first reported case of severe accidental hypothermia and thiamine deficiency responsible of heart failure in a malnourished child. Bi-ventricular systolic and diastolic dysfunctions both occurred after rewarming and refeeding. Further investigations are needed to clarify the effects of severe hypothermia on children's heart function. The possibility of beriberi as a cause of heart failure and adequate thiamine supplementation should be considered in all high-risk patients, including those with malnutrition. Refeeding syndrome requires careful management, including gradual correction of electrolyte imbalances and administration of a thiamine loading dose to prevent or correct refeeding-induced TD.

## Data Availability

All datasets generated for this study are included in the manuscript and/or the supplementary files.

## Ethics Statement

Written informed consent was obtained from the parent of the patient for the publication of this case report and any potentially identifying information/images.

## Author Contributions

AV concepted and designed the work, wrote, and structured the manuscript. AV, CD, SR, AW, MM, CJ, and DB revised the manuscript. AV, CD, SR, XB-P, DV, CV, and DB interpreted the data and managed the patient. All authors have approved the submitted version.

### Conflict of Interest Statement

The authors declare that the research was conducted in the absence of any commercial or financial relationships that could be construed as a potential conflict of interest.
